# Supplementary material for: A novel fasting mimetic (Mimio) creates fasting-like benefits to hunger control, oxidative stress, and cardiometabolic health in humans
Source: Sci Rep. 2026 Feb 20;16:7812. doi: 10.1038/s41598-026-38495-7 (PMC12953901; doi:10.1038/s41598-026-38495-7)
Supplement: Supplementary file 1 — Supplementary Material 1 [file 41598_2026_38495_MOESM1_ESM.docx]

**Supplemental Materials**

**Supplemental Table 1. Study Activities**

| **Protocol Activities** | **Screen^(a)^** | **Study Duration** | | | | | | | | | | |
| --- | --- | --- | --- | --- | --- | --- | --- | --- | --- | --- | --- | --- |
|  |  | **Randomize**  **and Ship^(b)^** | **Baseline** | **Study Product/Placebo Use Period**  **(8 weeks)** | | | | | | | | |
|  |  |  |  | **Week**  **1** | **Week**  **2** | **Week**  **3** | **Week**  **4** | **Week 5** | **Week 6** | **Week 7** | **Week 8** | **End of Study** |
|  | **Up to Day**  **-14** | **Up to Day 0** | **Day 1-14** | **Day 15–21** | **Day 22-28** | **Day 29-35** | **Day 36-42** | **Day 43-49** | **Day 50-56** | **Days 57-63** | **Day 64-70** | **Up to Day 77** |
| Informed Consent | X |  |  |  |  |  |  |  |  |  |  |  |
| Demographics | X |  |  |  |  |  |  |  |  |  |  |  |
| Medical History | X |  |  |  |  |  |  |  |  |  |  |  |
| BMI^(c^**^)^** | X |  |  |  |  |  |  |  |  |  |  |  |
| Food Frequency Questionnaire^(d^**^)^** | X |  |  |  |  |  |  |  |  |  |  |  |
| Eligibility Confirmation | X |  |  |  |  |  |  |  |  |  |  |  |
| Randomization^(e^**^)^** |  | X |  |  |  |  |  |  |  |  |  |  |
| Receipt of Product & Supplies Shipment |  | X |  |  |  |  |  |  |  |  |  |  |
| Blood Tests^(f^**^)^** |  |  | X |  |  |  |  |  |  |  | X | |
| CFQ^(g^**^)^** |  |  | WEEKLY | | | | | | | | |  |
| Weekly Questionnaire^(h^**^)^** |  |  | WEEKLY | | | | | | | | |  |
| TFEQ-18^(i^**^)^** |  |  | WEEKLY | | | | | | | | |  |
| Hunger, Satiety and Cravings Scale^(j^**^)^** |  |  | DAILY | | | | | | | | |  |
| Study Product/  Placebo Use ^(k^**^)^** |  |  |  | DAILY | | | | | | | |  |
| Adverse Event Survey^(l^**^)^** |  |  |  | WEEKLY | | | | | | | |  |
| Experience Survey |  |  |  |  |  |  |  |  |  |  |  | X |

| - 1. Screening occurred within 2 weeks prior to Randomization   2. Randomization and Study Product/Supplies Shipment occurred within 2 weeks prior to Baseline   3. Body Mass Index was calculated from self-reported weight and height measured within the last 6 months   4. Screening Food Frequency Questionnaire and Dietary Style   5. Study participants were randomized to one of 2 groups: (1) Mimio or (2) matching placebo   6. Blood sample collection at the participant’s local Quest laboratory for NMR lipoprofile, oxidized LDL, hsCRP, HbA1c, insulin and plasma glucose. Overnight fasting was required prior to collection. The tests were done within the 14-day baseline period and within 7 days after the last Mimio/placebo use day.   7. Cognitive Failures Questionnaire^23^   8. Weekly custom questionnaire with Likert-scale and frequency questions on sleep, stress, mood, energy, pain and gastrointestinal symptoms (i.e., flatulence, bloating, abdominal discomfort, stool consistency/regularity, constipation)   9. Three-factor Eating Questionnaire - 18 item^24^   10. 7-item custom Hunger, Satiety and Cravings Scale   11. Use of Mimio/placebo, 2 capsules daily with a glass of water, 30-60 mins before the first major meal   12. Participants answered an adverse event question weekly during the Mimio/placebo use period |
| --- |

**Supplemental Figure 1. Raw Lab Values Before and After.**


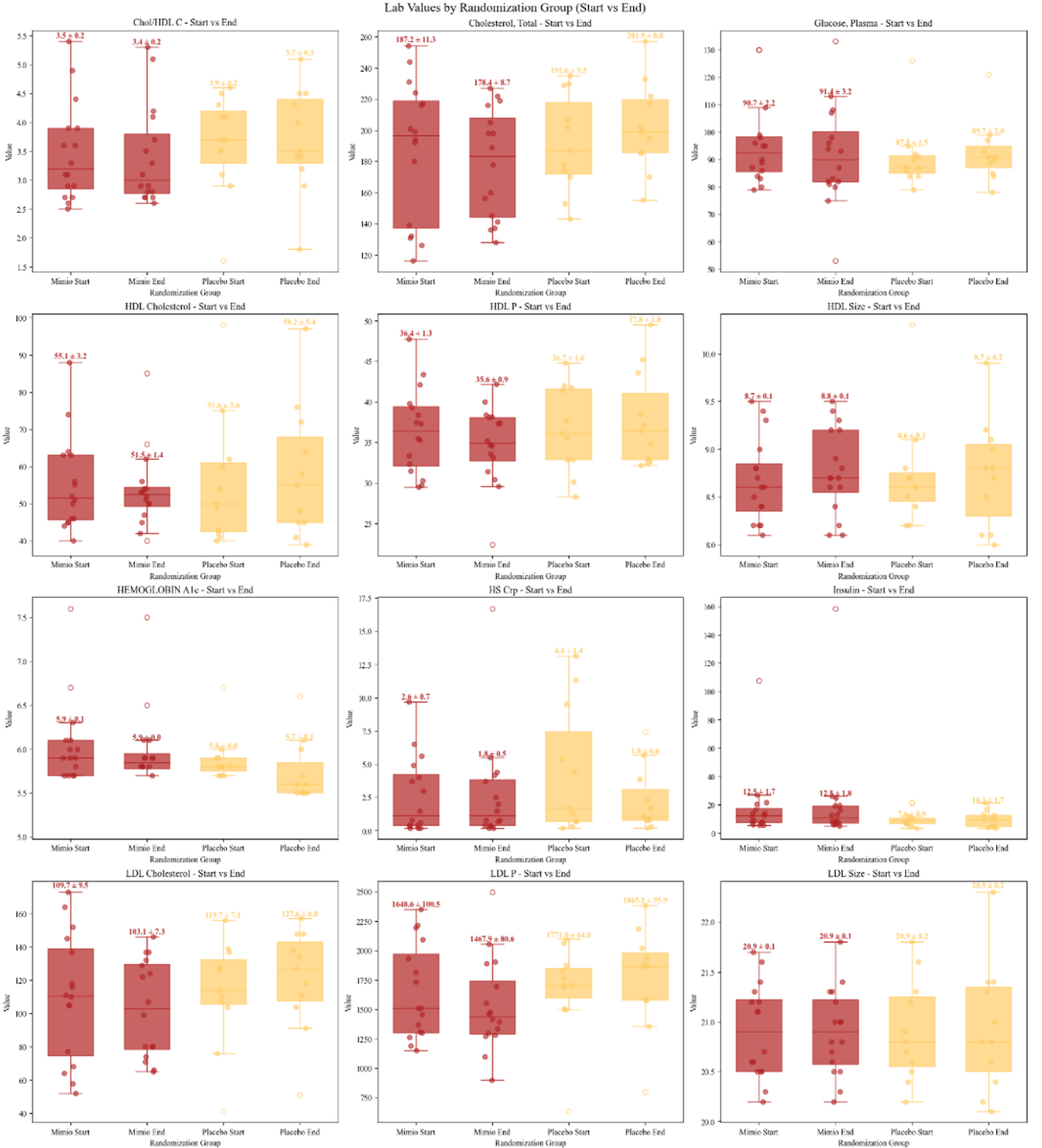


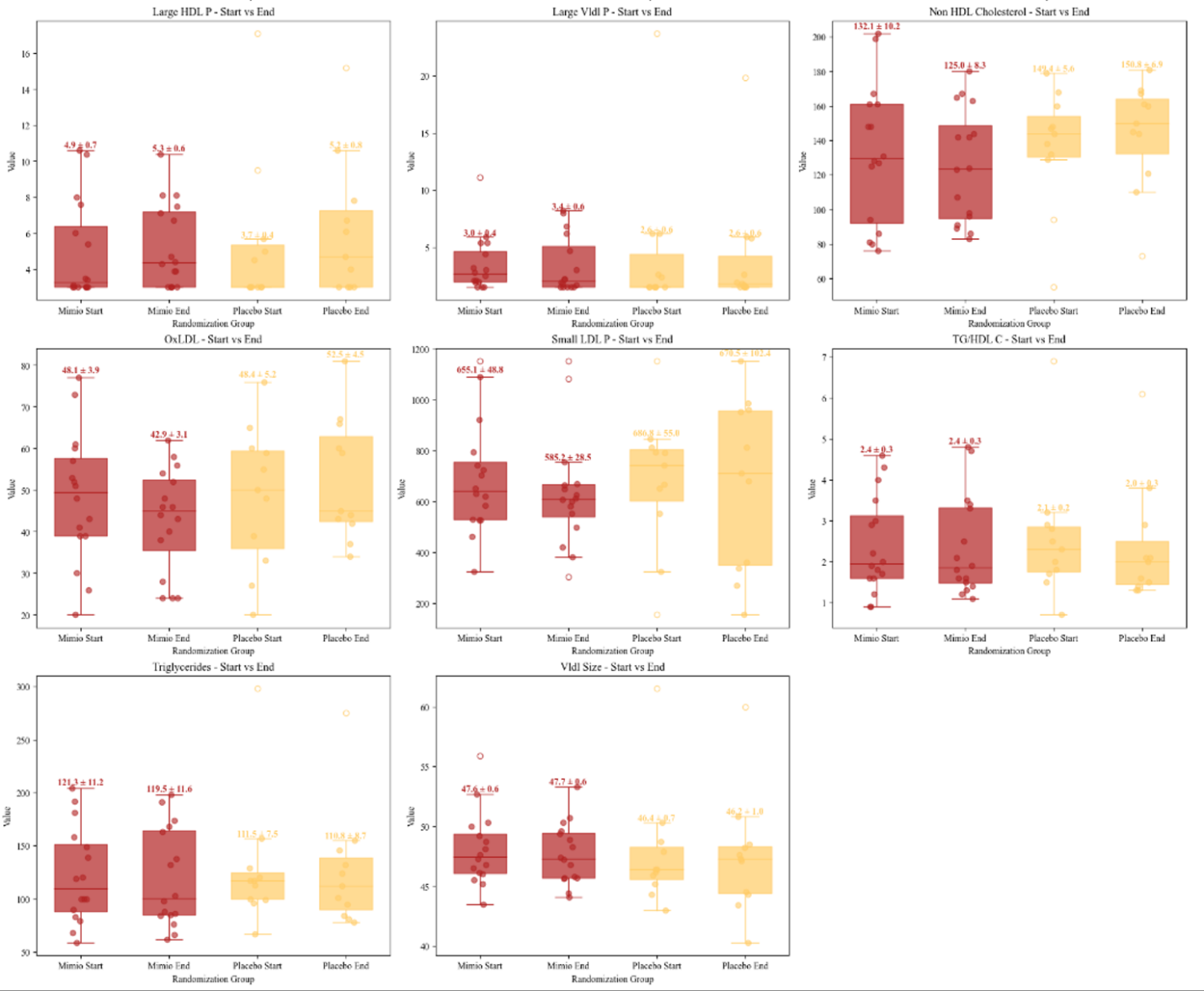


Supplemental Figure 1. Raw Laboratory Blood Values Before and After. Box and whisker plots with mean (SEM) depicted at the top of each whisker for Mimio (red) and placebo (yellow) participants at baseline and end of study (8 weeks). Individual dots represent individual participants’ data. Units can be found in main Table 3.

**Supplemental Table 2. Trends Over Time in Hunger & Satiety.**

| **Daily Metric** | **Mimio**  **Mean Trend (Mann-Kendall p-value)** | **Placebo**  **Mean Trend (Mann-Kendall p-value)** |
| --- | --- | --- |
| **Unhealthy Cravings** | Decreasing (p=3.8*10^-9^) | Decreasing (p=1.4*10**^-2^**) |
| **Eating Only When Hungry** | **Increasing (p=7.1*10^-3^)** | No Trend (p>0.05) |
| **Distraction From Cravings** | **Decreasing (p=7.7*10^-9^)** | No Trend (p>0.05) |
| **Postprandial Satiety** | **Increasing (p=1.2*10^-5^)** | No Trend (p>0.05) |
| **Mealtime Appetite** | **Decreasing (p=1.7*10^-12^)** | No Trend (p>0.05) |
| **Maximum Daily Hunger** | **Decreasing (5.6*10^-14^)** | No Trend (p>0.05) |
| **Overall Daily Hunger** | **Decreasing (2.5*10^-13^)** | No Trend (p>0.05) |
| **Composite Score** | **Decreasing (2.2*10^-16^)** | No Trend (p>0.05) |

**Supplemental Table 2**. *note that bolded rows indicate statistical difference between Mimio and Placebo. Baseline values did not differ statistically by group.

**Supplemental Table 3. % Improvers in Daily Hunger and Satiety**

| **Daily Metric** | **Mimio**  **% Improved** | **Placebo**  **% Improved** | **p-value** |
| --- | --- | --- | --- |
| **Unhealthy Cravings** | **65** | **73** | **0.74** |
| **Eating Only When Hungry** | **69** | **42** | **0.12** |
| **Distraction From Cravings** | **69** | **63** | **0.75** |
| **Postprandial Satiety** | **60** | **68** | **0.75** |
| **Mealtime Appetite** | **91** | **47** | **0.0025** |
| **Maximum Daily Hunger** | **87** | **58** | **0.04** |
| **Overall Daily Hunger** | **86** | **37** | **0.0011** |
| **Composite Score** | **74** | **53** | **0.2** |

****Metrics with statistically significant differences are shown in bold.***

**Supplemental Figure 2. Weekly Digestive Metrics at Week 8.**

**
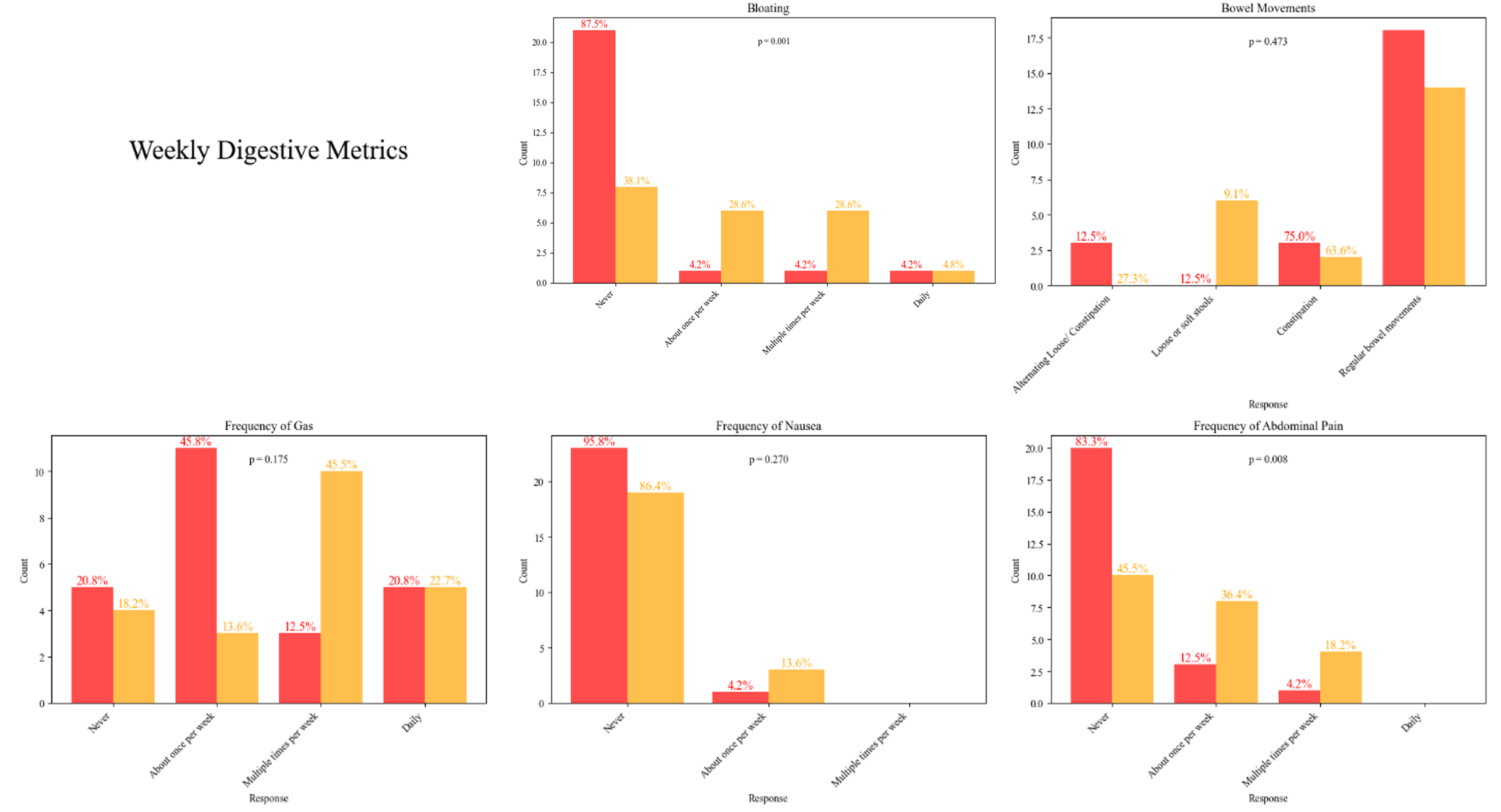
**

Supplemental Figure 2. Weekly Digestive Metrics at Week 8. Distribution of categorical responses for Mimio (red) and placebo (yellow) participants’ bloating, bowel movement type, frequency of gas, frequency of nausea and frequency of abdominal pain. Percentages represent the percent of that cohort that gave a given response. P-values represent Mann-Whitney U tests for difference in distributions between Mimio and placebo.

**Supplemental Figure 3. Other Weekly Metrics at Week 8**


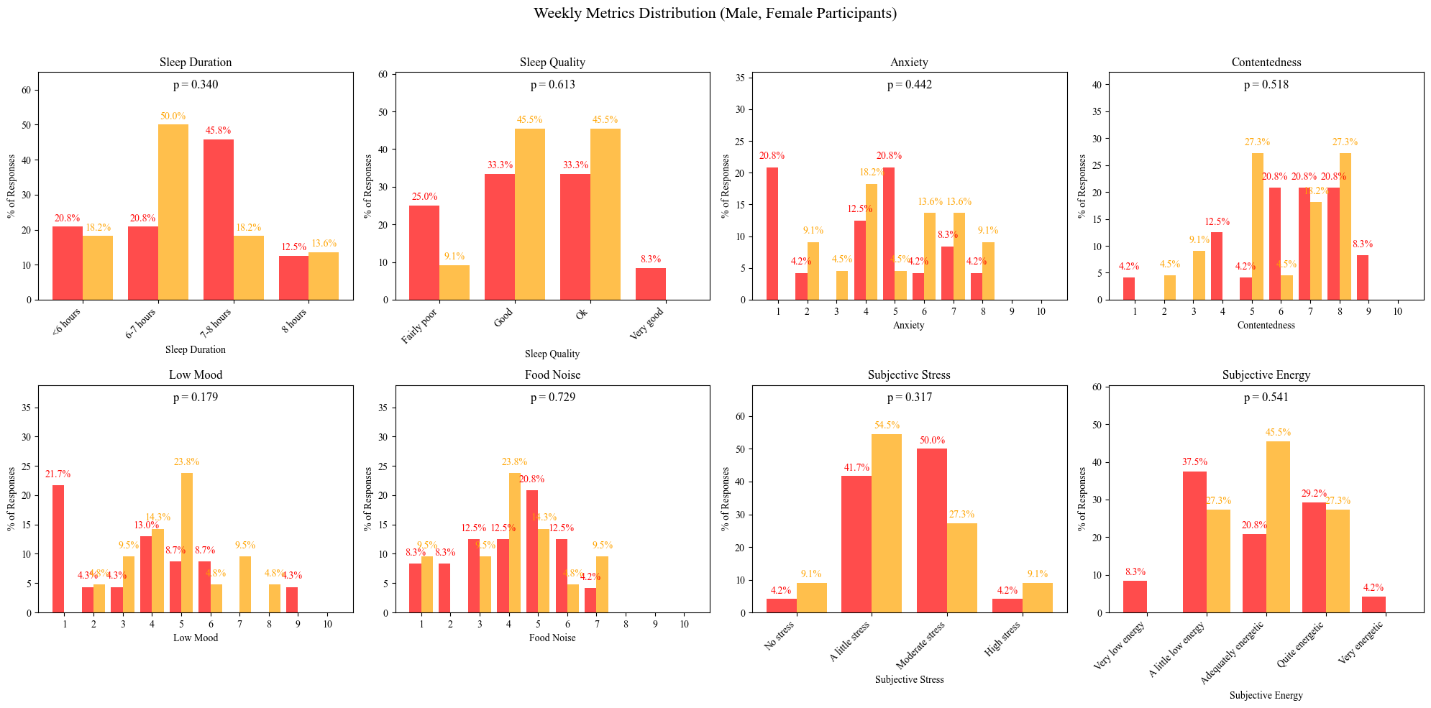


Supplemental Figure 3. Other Weekly Metrics at Week 8. Distribution of categorical responses for Mimio (red) and placebo (yellow) participants’ sleep duration, sleep quality, anxiety, contentedness, low mood, food noise, subjective stress and subjective energy. Percentages represent the percent of that cohort that gave a given response. P-values represent Mann-Whitney U tests for difference in distributions between Mimio and placebo.

**Adverse Events.** 6 AEs occurred during the study, 11 in the Mimio group and 10 in the Placebo group. Only 1 AE, a case of mild diarrhea, occurred during product use in the Mimio group and was considered possibly attributable to the intervention. By comparison, 5 digestive AEs occurred in the Placebo group. Please see Supplemental Table 4 for all categorized AEs. There were 2 SAEs unrelated to product use (counted under “Other”). One was a case of cellulitis that required hospitalization and IV medication in the Mimio group. The other was a heart attack in the placebo group. 2 additional AEs were categorized as “Other”, and none were attributed to the study intervention. These were a recurrence of leg pain in the Mimio group and a case of lightheadedness in the placebo group.

**Supplemental Table 4. Adverse Events.**

| **AE Type** | **Mimio** | **Placebo** |
| --- | --- | --- |
| **Digestive** | **1** | **5** |
| **Diarrhea** | **1** | **3** |
| **Stomach Pain** | **0** | **2** |
| **Gas** | **0** | **1** |
| **Headache** | **1** | **0** |
| **Cold or Flu** | **2** | **3** |
| **Allergy** | **0** | **1** |
| **Other** | **2** | **2** |

**Supplemental Figure 4. Three Factor Eating Questionnaire (TFEQ-18) Score Change at Week 8.**


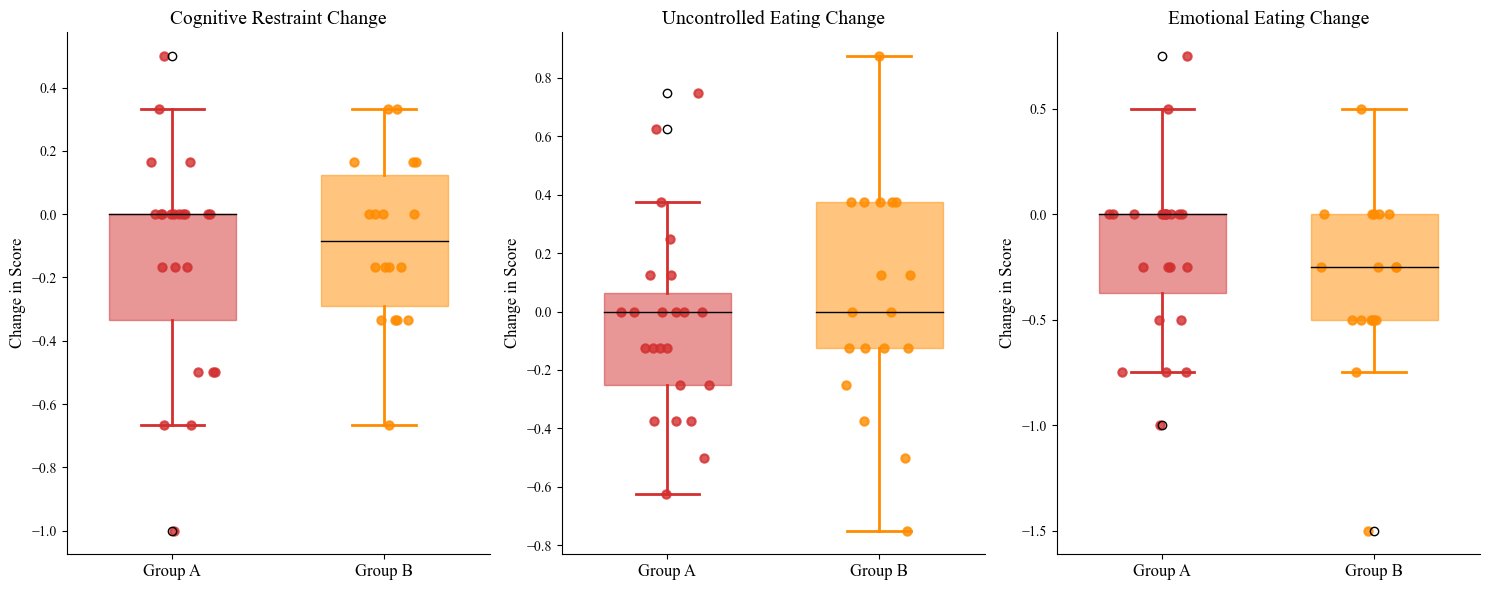


Supplemental Figure 4. Three Factor Eating Questionnaire (TFEQ-18) Score Change at Week 8. Box and whisker plots of change from baseline TFEQ-18 score to week 8 score in Mimio (red) and placebo (yellow) participants. Individual dots represent individual participants’ data.

**Supplemental Figure 5. Three Factor Eating Questionnaire (TFEQ-18) Scores Over Time**


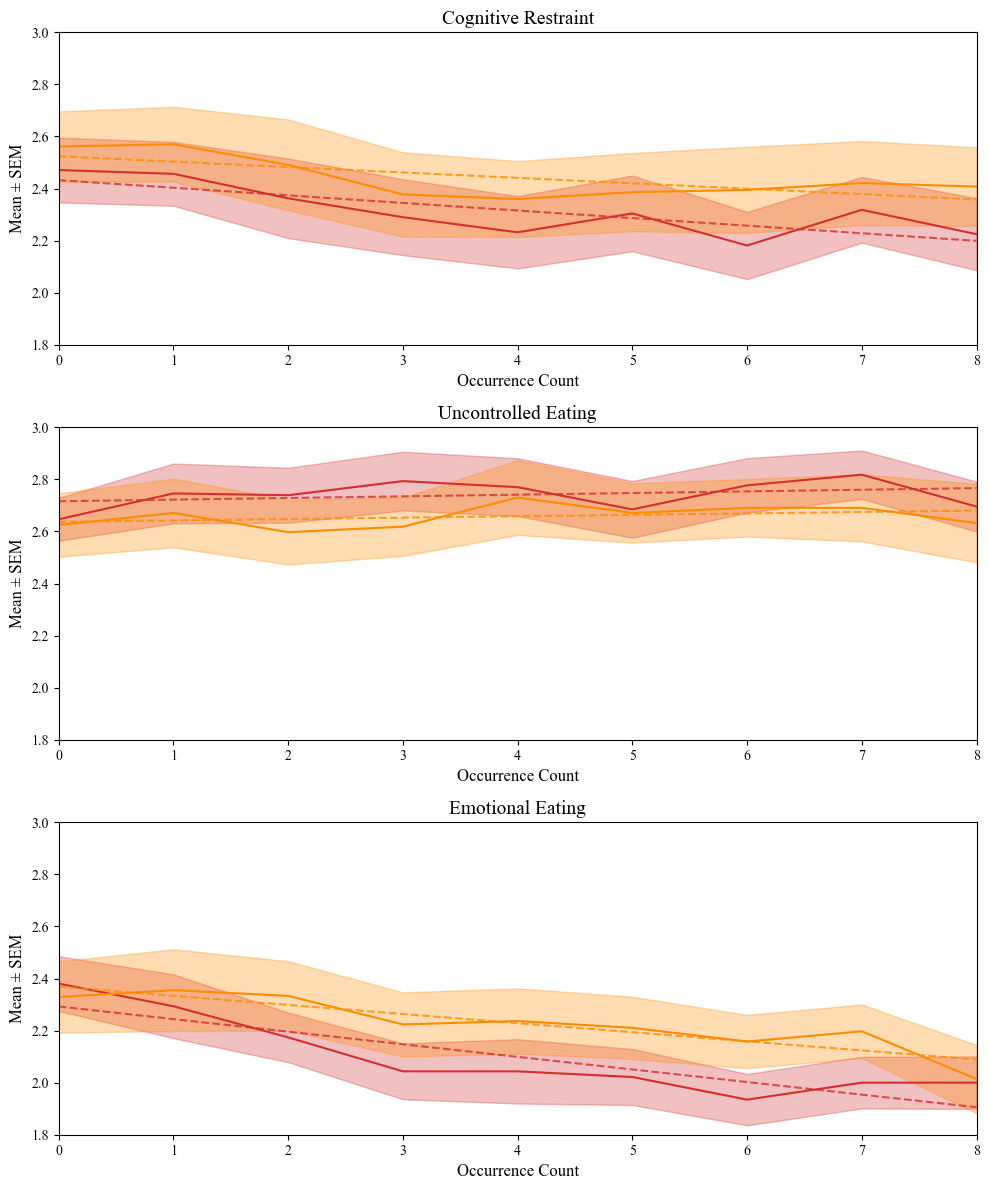


Supplemental Figure 5. TFEQ-18 Scores Over Time. Mean with shaded SEM scores are shown from Week 0 (baseline) through 8 weeks of product or placebo use. Cognitive restraint (top), uncontrolled eating (middle) and emotional eating (bottom) components of the TFEQ-18 are shown separately. Mimio is shown in red and placebo in yellow. Trend lines are graphed for each subcomponent.

**Supplemental Figure 6. Cognitive Failures Questionnaire**


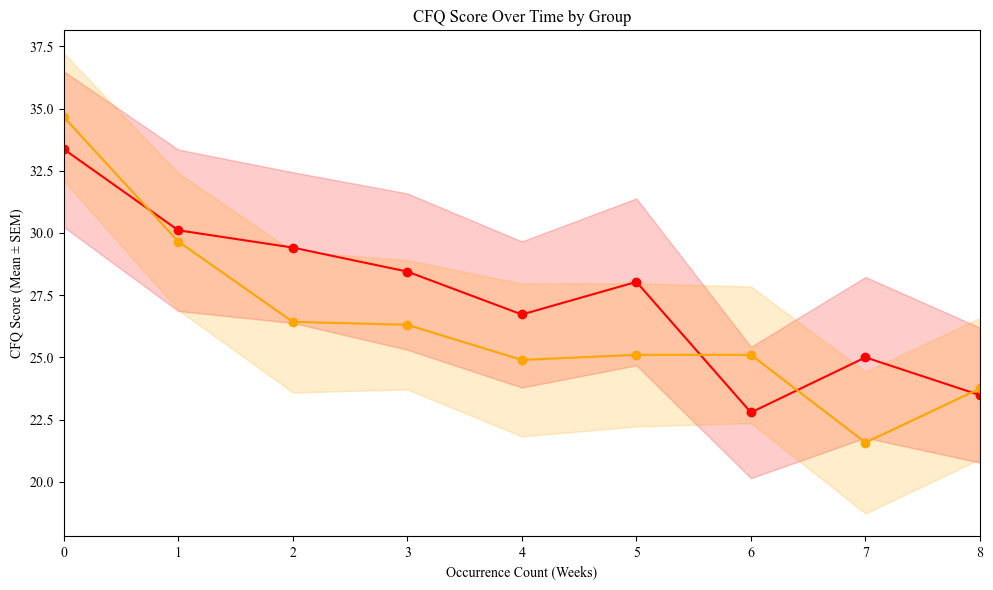


Supplemental Figure 6. Cognitive Failures Questionnaire. Mean with shaded SEM weekly CFQ score in Mimio (red) and placebo (yellow) from baseline (0) to week 8 of Mimio/placebo use.
